# Aging, cell senescence, the pathogenesis and targeted therapies of intervertebral disc degeneration

**DOI:** 10.3389/fphar.2023.1172920

**Published:** 2023-05-05

**Authors:** Jiongnan Xu, Ting Shao, Jianfen Lou, Jun Zhang, Chen Xia

**Affiliations:** ^1^ Center for Plastic and Reconstructive Surgery, Department of Orthopedics, Zhejiang Provincial People’s Hospital, Affiliated People’s Hospital Hangzhou Medical College, Hangzhou, China; ^2^ The Second School of Clinical Medicine, Zhejiang Chinese Medical University, Hangzhou, China; ^3^ Department of Orthopedics, Zhejiang Provincial People’s Hospital Bijie Hospital, Bijie, Guizhou, China

**Keywords:** aging, cell senescence, pathogensis, targeted therapies of aging, intervertebral disc degeneration

## Abstract

Intervertebral disc degeneration (IVDD) refers to the aging and degenerative diseases of intervertebral disc components such as nucleus pulposus, annulus fibrosus, and cartilage endplate, and is the main cause of chronic low back pain. Over the past few years, many researchers around the world concerned that the degeneration of nucleus pulposus (NP) cells plays the main role in IVDD. The degeneration of NP cells is caused by a series of pathological processes, including oxidative stress, inflammatory response, apoptosis, abnormal proliferation, and autophagy. Interestingly, many studies have found a close relationship between the senescence of NP cells and the progression of NP degeneration. The classical aging pathways also have been confirmed to be involved in the pathological process of IVDD. Moreover, several anti-aging drugs have been used to treat IVDD by inhibiting NP cells senescence, such as proanthocyanidins, resveratrol and bone morphogenetic protein 2. Therefore, this article will systematically list and discuss aging, cell senescence, the pathogenesis and targeted therapies of IVDD, in order to provide new ideas for the treatment of IVDD in the future.

## 1 Introduction

Low back pain (LBP) is one of the most common clinical health problems, experienced by people of all ages, and as the number one cause of disability worldwide, it imposes significant healthcare costs on society (Hoy et al., 2010; Vos et al., 2015; Maher et al., 2017; Clark and Horton, 2018; Hartvigsen et al., 2018). As the most important cause of LBP, intervertebral disc degeneration (IVDD) refers to the pathophysiological process of the progressive structural failure of intervertebral disc (IVD) (Adams and Roughley, 2006; Zhang et al., 2021c). The pathogenesis of IVDD is complex and mainly includes aging, inflammation, oxidative stress, mechanical stress and genetic factors (Sharma, 2018; Li et al., 2020; Wen et al., 2022; Xin et al., 2022). All of these factors promote degradation of the IVD extracellular matrix (ECM), leading to dysfunction and structural damage of the IVD (Risbud and Shapiro, 2014; Wang D. et al., 2020; Zhang G. Z. et al., 2021). Nevertheless, the mechanisms of IVDD are still not well established. An increasing number of researchers are dedicated to clarify the pathogenesis of IDD.

The IVD consists of mainly three parts: the nucleus pulposus (NP), annulus fibrosus (AF), and two cartilaginous endplates (CEP) (Clouet et al., 2009). The NP consists of NP cells and ECM, which mainly includes type II collagen (Col II) and proteoglycans (Pattappa et al., 2012; Rustenburg et al., 2018). The dynamic balance of ECM composition and content determines the structural and functional integrity of the NP(Xin et al., 2022). ECM catabolic enzymes show high expression in degenerating NP cells, such as matrix metalloproteinases (MMPs) and a disintegrin and metalloprotease with thrombospondin motifs (ADAMTS), ultimately leading to ECM degradation (Nagase and Kashiwagi, 2003; Wang et al., 2015; Sun et al., 2022). Therefore, we should pay attention to the dynamic balance of ECM anabolism and catabolism, which is the key to the treatment of IVDD.

Studies have found that the prevalence of IVDD increases dramatically with age, with a 50% difference in the prevalence of intervertebral disc disease between people in their 50s and 70s (Miller et al., 1988). Histological studies have shown that IVD cells begin to senesce as early as the second decade of life (Dowdell et al., 2017). New evidence from clinical and animal model studies suggests that senescent IVD Cells, particularly senescent NP Cells, accumulate in aging and degenerating IVD, which is considered a new hallmark and major cause of IVDD (Dowdell et al., 2017; Zhang K. et al., 2021; Sun et al., 2021). The role of senescent NP Cells in IVDD has become clearer in recent years, and its related literature is growing. The causative mechanisms of aging as a major cause of IVDD deserve to be explored and studied.

Cellular senescence is characterized by irreversible cell cycle arrest and senescence-associated secretory phenotype (SASP). Senescent cells (SCs) with SASP secrete numerous factors such as pro-inflammatory cytokines and chemokines, growth regulators, angiogenic factors, and MMP(Patil et al., 2019b; Gorgoulis et al., 2019). In studies over the past few years, it has been found that SASP produced by SCs in IVDCs can disrupt the metabolic balance in the ECM(Wang and Tissenbaum, 2006; Wu Y. et al., 2022). It was shown that the expression levels of ECM proteases (MMP-13, ADAMTS-4 and ADAMTS-5) were significantly increased in aging IVD, along with degradation of more Col II and proteoglycan (Wang and Tissenbaum, 2006; Bedore et al., 2014). Therefore, it is important to know the specific pathways of how aging occurs in IVD Cells, and to provide ideas for targeted therapies for IVDD. Currently recognized classical molecular signaling pathways of cellular senescence include mTOR, AMPK, NF-κB, Sirtuins, P16, and p53 (Rayess et al., 2012; Osorio et al., 2016; Van de Ven et al., 2017; Ou and Schumacher, 2018; Stallone et al., 2019; Ge et al., 2022). Based on the above-mentioned signaling pathways, there have been many studies investigating the effects of cellular senescence in IVDD (Zhang et al., 2019; Che et al., 2020; Yurube et al., 2020; Shao et al., 2021). In addition, anti-aging drugs have been studied for the treatment of IVDD, such as proanthocyanidins, resveratrol, and bone morphogenetic protein 2 (Tan et al., 2019; Chen H.-W. et al., 2022; Liu et al., 2022).

This review provides an overview of the relationship between aging in IVD Cells and IVDD, summarizes the research progress of some classical pathways of aging in IVDD and strategies for anti-cellular aging, and discusses the future direction of clinical application.

## 2 Mechanism of intervertebral disc degeneration

The IVD consists of three layers: AF, central NP and CEP (Colombini et al., 2008). AF is mainly used to protect the NP from freeing from the IVD when the spine is under high load. In IVD, nutrition penetrates into the NP through the CEP, so the NP, as a tissue lacking blood vessels and nerves, is very slow and difficult to repair after injury (Sampara et al., 2018). Although the NP Cells account for only 1% of the volume of the IVD, they play the most important role in the physiology and biomechanics of the IVD (Vo et al., 2016; Lu et al., 2020). NP Cells regulate the metabolism of ECM and together constitute NP. The ECM of the NP consists mainly of Col II, and proteoglycans, which are used to counteract and transmit the axial pressure of the spine during loading (Chen et al., 2020; Xi et al., 2020). ECM of NP is important to maintain the structural and functional integrity of the IVD and to help prevent IVDD (Qiu et al., 2019). Studies have shown that the imbalance between ECM anabolism and catabolism play an important role in IVDD, ultimately leading to the loss of proteoglycans and hydration (Sampara et al., 2018).

**FIGURE 1:**
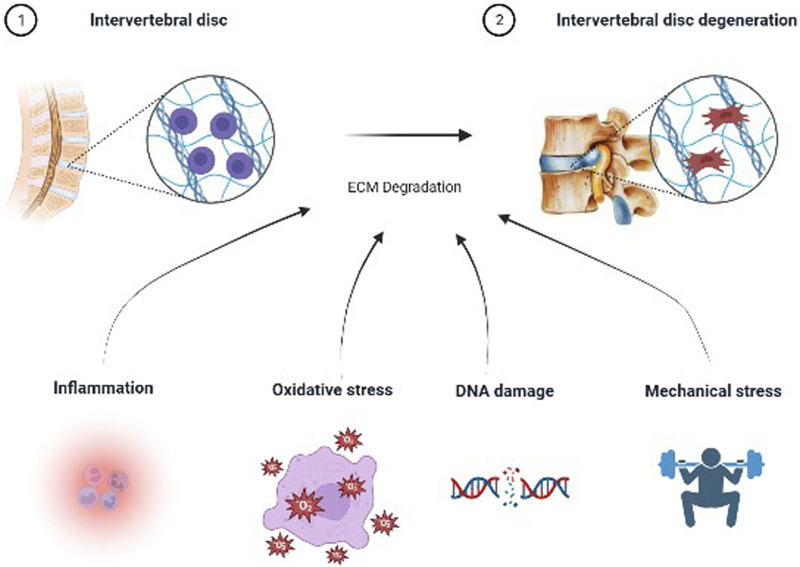
Etiology of intervertebral disc degeneration.

IVDD, a common and complex disease, is associated with multiple pathological factors. Several studies have shown that multiple pathological factors such as inflammation, oxidative stress, mitochondrial dysfunction, and abnormal mechanical loading are involved in the development of IVDD (Feng et al., 2016; Ao et al., 2019; Chen et al., 2020; Zhang et al., 2020). These pathological factors lead to the degradation of ECM through various pathways and finally cause IVDD (Figure 1). Studies have demonstrated that inflammatory mediators such as IL-1β disrupt the metabolic homeostasis of the ECM(Wang L. et al., 2020). In recent years, there is increasing evidence that mitochondrial dysfunction increases the production of reactive oxygen species (ROS), and that excess ROS activate IVDD by regulating matrix metabolism, pro-inflammatory phenotype, autophagy, senescence, and apoptosis of IVDC (Davalli et al., 2016; Feng et al., 2017b; Kang et al., 2020; Cao et al., 2022). Studies have demonstrated that when the IVD is subjected to abnormal mechanical stress, it promotes the degradation of ECM by IVDCs and the development of IVDD (Vergroesen et al., 2015).

## 3 Aging, cell senescence

### 3.1 Cellular senescence

**FIGURE 2:**
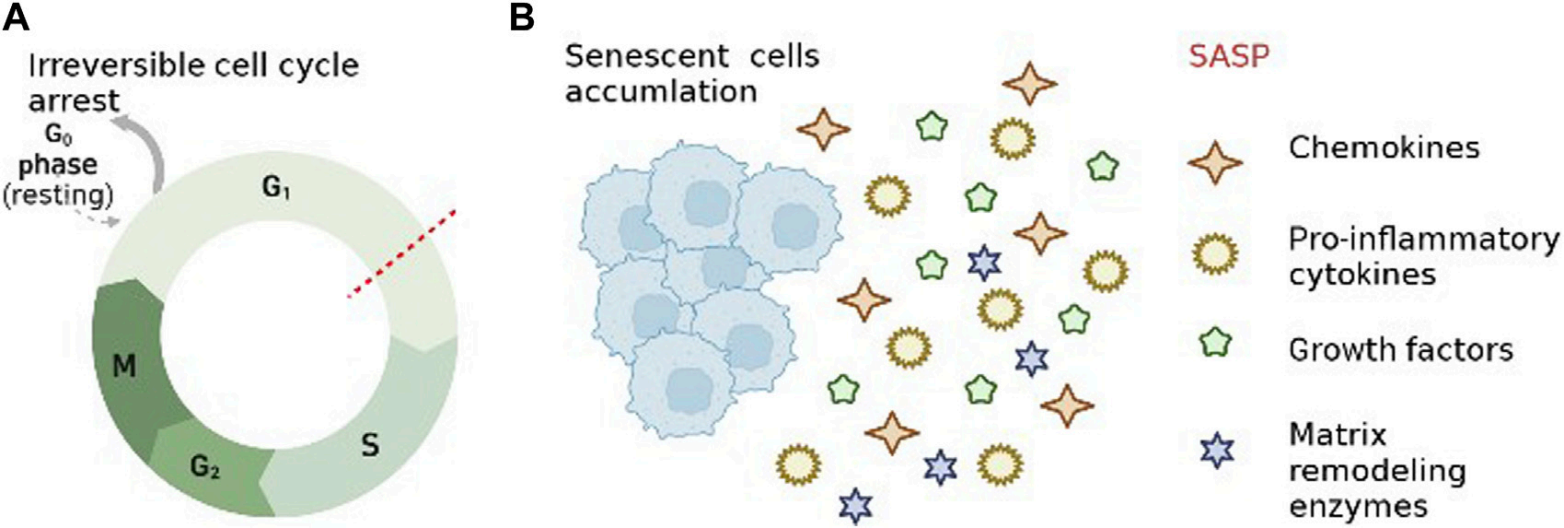
Characteristics of cellular senescence. **(A)**. Irreversible cell cycle arrest. **(B)**. senescence-associated secretory phenotype.

Aging is a process in which the tissues and organs of an organism undergo irreversible functional decline and weakening of the regulation of internal environmental homeostasis as they age, and gradually tend to die (Kennedy et al., 2014; Fulop et al., 2019). Hayflick et al. found that human fibroblasts exhibit a limited ability to divide cells before entering irreversible growth arrest, known as replicating aging. Thus they hypothesized that the gradual loss of cell proliferation eventually leads to tissue aging (Hayflick and Moorhead, 1961). Cellular senescence is a state of irreversible cell cycle arrest in cells driven by continuous exogenous and endogenous stress and injury (Wu Y. et al., 2022) (Figure 2 A). SCs, a product of cellular aging process, have special biological characteristics and functions, and are one of the important causes of impaired tissue regeneration, chronic aging-related diseases and organismal aging. There is no single marker with absolute specificity for senescent cells. The combination of cytoplasmic (e.g., senescence-associated β-Galactosidase (SA-β-Gal), lipofuscin), nuclear (e.g., p16^INK4A^, p21^WAF1/Cip1^, Ki-67) is now commonly used for the detection of SCs(Childs et al., 2015; Wu Y. et al., 2022). Currently, microscopic imaging is the method of choice for detecting aging. In addition, single-cell assays, such as immunostaining, *in situ* hybridization, and multicolor (imaging) flow cytometry, are also used to detect SCs(Gorgoulis et al., 2019). A quantitative study of SCs by Anat Biran et al. found significantly more SCs in older mice than in younger mice (Biran et al., 2017). The accumulation of SCs with age has a negative impact since the immune system fails to completely remove them (Kowald et al., 2020). Short-term cellular senescence is essentially harmless because the immune system will completely remove it, however long-term cellular senescence can lead to inflammation and disease (Herranz and Gil, 2018). In addition to irreversible cell cycle arrest, changes in chromatin, gene expression, organelles and cell morphology are also characteristic of cellular senescence (Gorgoulis et al., 2019). Meanwhile the extrinsic activity of SCs is extensively associated with the activation of SASP, which enhances the effects of intrinsic cell proliferation arrest and leads to impaired tissue regeneration, age-related chronic diseases and organismal aging (Di Micco et al., 2021).

### 3.2 SASP

As another feature of cellular senescence, SASP refers to the state of senescent cells that secrete different chemokines, pro-inflammatory cytokines, growth factors and matrix remodeling enzymes (Shelton et al., 1999; Rodier et al., 2009) (Figure 2B). SASP-related proteins, such as TNF-α, IL-6, MMPs, monocyte chemotactic protein-1 and IGF binding proteins, increase over time with aging in multiple tissues (Freund et al., 2010). It has been shown that the secretion of SCs is constantly changing during the development of cellular senescence because SASP is regulated at multiple levels (transcription, translation, and secretion) (Hoare et al., 2016; Malaquin et al., 2016). Furthermore SASP induces neighboring cells to senesce and amplify SASP by autocrine and paracrine means (Herranz and Gil, 2018; Patil et al., 2018). The inflammatory microenvironment generated by SASP via autocrine and paracrine secretion has been identified as a key step in the development of age-related diseases, such as IVDD, osteoarthritis, Osteoporosis (Birch et al., 2015; Feng et al., 2016; Jeon et al., 2017). The inflammatory mediators of SASP are powerful drivers of tumor progression, and early studies have shown that SASP can increase tumor angiogenesis and promote malignant cell proliferation and metastasis (Krtolica et al., 2001; Coppé et al., 2006). SASP is a double-edged sword in specific cases (Herranz and Gil, 2018). On the one hand, it accelerates cellular senescence through paracrine secretion (inducing non-malignantly proliferating neighboring cells to undergo senescence), and on the other hand, it can reinforce senescent growth arrest through autocrine secretion, such as suppression of senescent tumors (Hubackova et al., 2012; Nelson et al., 2012; Acosta et al., 2013).

## 4 Effects of senescent cells on intervertebral disc cells

To date, IVDD is known to be driven by loss of IVD Cells, anabolic factor deficiency, and activation of matrix degrading enzymes (Risbud and Shapiro, 2014; Wang et al., 2016). The proportion of senescent NP Cells was significantly increased in degenerated IVDs, and the lifespan of senescent NP Cells was shorter compared to normal NP Cells (Jeong et al., 2014). Compared to younger patients, NP samples from older IVDD patients showed a higher proportion of senescent NP Cells, higher p53 and p21 expression, higher SA-β-Gal activity and lower telomere length (Kim et al., 2009). Senescent NP Cells in SASP secrete a variety of substances including cytokines, chemokines, growth factors and ECM proteases, among which ECM proteases (MMPs and ADAMTS) cause excessive breakdown of ECM and the microenvironment of NP, thus greatly contributing to the development of IVDD (Wang and Tissenbaum, 2006; Bedore et al., 2014). Therefore, it is particularly crucial to investigate the pathological factors that lead to NP Cells senescence.

### 4.1 Etiology of nucleus pulposus cellular senescence

**FIGURE 3:**
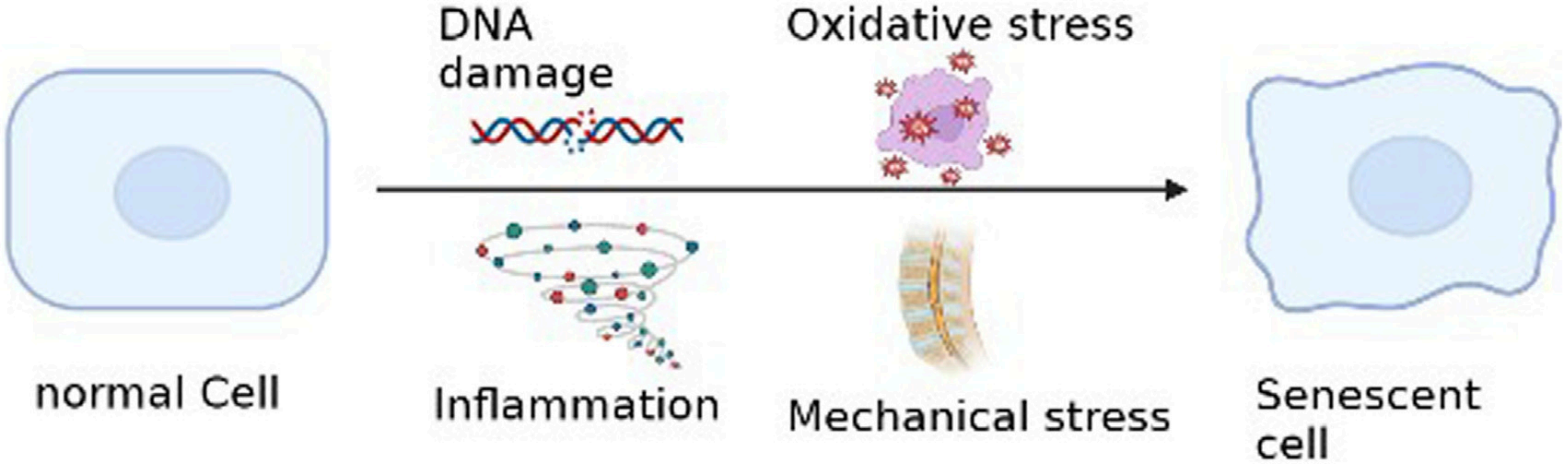
Etiology of intervertebral disc cell senescence.

There are many causes of cellular senescence, and we summarize the most important pathological factors that lead to NP Cells senescence. (Figure 3).

#### 4.1.1 Inflammation

Chronic inflammation, considered as a sign of aging, is a risk factor for age-related diseases (Franceschi et al., 2000; López-Otín et al., 2013; Ferrucci and Fabbri, 2018). During IVDD pathology, NP Cells produce excess inflammatory factors (such as IL-1β and TNF-α) that trigger the subsequent pathogenic process of aging (Le Maitre et al., 2005; Le Maitre et al., 2007).

D Purmerrur et al. performed SA-β-Gal staining and qPCR assay to evaluate the senescence and ECM homeostasis (Purmessur et al., 2013). The data showed that the proportion of positive SA-β-Gal stained cells was significantly greater in the TNF-α group than in the control group, moreover, the gene expression of aggrecan, type Ⅰ collagen (Col Ⅰ), and Col Ⅱ was significantly downregulated in the NP. Therefore, the author hypothesized that TNF-α may induce senescence and disrupt the microenvironmental homeostasis of IVD Cells through some mechanism. In another study, the authors explored the differences between senescence and puncture-induced IVDD models in rats (He et al., 2022). The relationship between inflammation and senescence in NP Cells was studied and evaluated by immunohistochemical detection of changes in ECM (Col I, Col II) and detection of inflammation-related indicators (TNF-α). Immunohistological results showed a higher percentage of TNF-α positive cells in the senescent-group compared to the punctured-group. The investigators used alcian blue and safranin o staining to observe the composition and content of ECM in IVD. The histological results revealed a decrease in the stained area of IVD in both the senescent and punctured groups, and the punctured group had the smallest stained area of IVD. Aging-induced and puncture-induced IVDD manifestations were different, with chronic inflammatory indicators rising in the senescent group and the punctured group suffering from severe ECM loss. TNF-α induced inflammation plays a great role in senescence of NP Cells and ECM homeostasis. Kang et al. used TNF-α and IL-18 in mouse NP to mimic the inflammatory environment and construct an IVDD model (Li et al., 2019). They found a higher proportion of NP Cells senescence and senescence markers (P16 and p53) in treated NP. The investigators also used the CCK-8 assay to assess cell proliferation and found that TNF-α-induced NP Cells had significantly lower proliferative capacity. Moreover, the result of flow cytometry of NP Cells revealed a significant increase in the G0/G1 phase and a significant decrease in the S phase. These data mean inflammatory factors induce senescence in NP Cells, as evidenced by reduced cell proliferation capacity and cell cycle arrest.

The specific pathogenesis of obesity-induced IVDD has been under investigation. Interestingly, Sunli Hu et al. isolated NP tissue from patients with different levels of IVDD and evaluated the proportion of senescent NP Cells and the amount of chemerin in the NP(Hu et al., 2020). They found that the levels of senescent NP Cells and Chemerin increased significantly with increasing degree of IVDD. Thus, Hu et al. speculated that aging of NP Cells may be associated with chemerin. Several previous studies have shown that the NF-kB pathway plays an important role in obesity-related inflammation. Therefore, Hu detected the expression levels of p65 and AKT in chemerin induced senescent NP Cells. Western blotting showed that chemerin stimulation significantly increased the expression levels of p65 and AKT. And the investigators found that the use of Lv-shTLR4 reversed chemerin-induced activation of the NF-κB signaling pathway, and Lv-shCMKLR1 reduced AKT phosphorylation and SA-β-Gal-positive NP Cells. Therefore, the authors concluded that chemerin activates the NF-κB signaling pathway through its receptors CMKLR1 and TLR4, releasing inflammatory mediators, leading to cellular senescence.

Apart from this, according to previous studies, other pro-inflammatory mediators, including, IL-2, IL-4, IL-8, IL-10, IL-17, IFN-γ, chemokines and prostaglandin 2 promote aging of NP Cells, which leads to the development of IVDD (Wuertz and Haglund, 2013; Risbud and Shapiro, 2014; Ohtori et al., 2015). However, their role of IVDD still needs to be explored.

#### 4.1.2 Mitochondrial dysfunction and oxidative stress

Mitochondria, an evolutionary product of aerobic bacterial invasion of eukaryotic cells a billion years ago, have a separate genome for the synthesis of ATP. As a complex organelle, mitochondria are not only energy workstations, but also regulate cellular senescence (Zhang et al., 2022). The most classical mitochondria-induced senescence-related pathways available are P38/MAPK, P16-RPB and P53 (Van Deursen, 2014). Subsequently, more and more researchers identified mitochondrial dysfunction as being associated with NP Cells cellular senescence. A study by Xiaolong Xu et al. investigated the correlation between senescence and mitochondrial dysfunction in NP Cells (Xu et al., 2019). Progerin, a truncated unprocessed lamin A protein, causes tissue aging and degeneration. Xu found that progerin levels increased with IVDD progression in human and rats NP tissue. Researchers infected rat NP Cells with mCherry-progerin-expressing or vector-only-expressing lentiviruses. They found that progerin expression decreased the levels of the nuclear structure-associated proteins lamin B1 and lamina-associated polypeptide2, and the extent of lysine 27 trimethylation on heterochromatin-associated histone 3, but increased the expression of serine-139 phosphorylated H2AX and the proportion of SA-β-Gal-positive cells. At the same time, the progerin group exhibited elevated ROS levels, disruption of mitochondrial membrane potential, decreased ATP production, and reduced activity of mitochondrial complex enzymes compared to the vector control group. Overall, these data clearly indicate that upregulation of progerin expression in NP cells induces severe oxidative stress and mitochondrial dysfunction, triggering senescence-related defects and apoptosis *in vitro*. To explore the link between progerin overexpression and IVDD progression, Xu et al. constructed LmnaG609G knock-in mice (progerin overaccumulation). And mouse tissue samples were analyzed histologically and levels of progerin were assessed using Western blotting. Mitochondrial function was measured by analyzing mitochondrial membrane potential and ATP levels. The results showed that excessive progerin expression *in vivo* induced NP senescence and mitochondrial dysfunction. Overexpression of progerin in NP Cells induces mitochondrial dysfunction, leading to cellular senescence.

ROS accumulate in large amounts as a byproduct of ATP synthesis due to mitochondrial dysfunction. The large accumulation of ROS in NP Cells can also leads to senescence (Feng et al., 2017a). Wang et al. showed that oxidative stress can induce mitochondrial dysfunction and exacerbate the aging of NP Cells (Wang Y. et al., 2018). Phosphatase and Tensin homolog-induced putative kinase protein 1 is a mitochondria-targeted serine/threonine kinase that protects against mitochondrial dysfunction and mitochondrial quality control by activating PINK1/Parkin-mediated mitophagy. The investigators induced oxidative stress in human NP Cells by H2O2. And the senescence of NP Cells was detected after targeted silencing of PINK1 by short hairpin RNA. It was found that downregulation of PINK1 in H2O2-treated NP Cells resulted in a significant increase in the proportion of senescent NP Cells. PINK1 plays a protective role in the clearance of damaged mitochondria and mitigation of NP Cells senescence under oxidative stress by a mechanism related to the regulation of mitochondrial autophagy. Liang et al. conducted a similar study and further confirmed that oxidative stress promotes aging in NP Cells (Kang et al., 2019). The investigators used tert-butyl hydroperoxide (TBHP) to induce oxidative stress and mitochondrial dysfunction in rat NP Cells. The results show TBHP increase the levels of Bax, cleaved caspase-3, cleaved caspase-9 and cytoplasmic cytochrome c and decrease the levels of Bcl-2 and mitochondrial cytochrome c in NP Cells. However, curcumin pretreatment attenuated these changes and reduced the proportion of senescence in NP Cells. Moreover, Liang also found higher phosphorylation level of AMPK and ULK1 and lower phosphorylation of mTOR in the western blotting results. All these data suggest that the AMPK/mTOR/ULK1 pathway is involved in oxidative stress related NP cells senescence.

#### 4.1.3 Mechanical stress

Abnormal mechanical stress is an important risk factor for IVDD, and cellular senescence is a pathological alteration of IVDD. However, the molecular mechanisms of mechanical stress-induced cellular senescence have not been fully elucidated.

In a study addressing mechanical stress and aging of NP Cells, Qiujuan Xing et al. constructed models of abnormal stress by performing forelimb amputations on mice (Xing et al., 2010). Quantitative immunohistochemical analysis showed that the mRNA levels of p16INK4a, RB, PTEN, p27KIP, p19ARF and RAGE were upregulated, and the mRNA of p21 was significantly downregulated. The highest levels of SA-β-GaL activity and significant increased cell cycle protein D1 mRNA levels were detected in rats with 9 months of amputation. The researchers hypothesized that the abnormal stress caused by amputation affected the function of cell cycle regulatory proteins and accelerated the aging of NP Cells. In another study, Yiyang Wang et al. found that overload mechanical compression induces oxidative stress, mitochondrial dysfunction and aging in human NP Cells (Wang et al., 2021). The investigators explored the association between mechanical stress and aging of NP Cells by providing different mechanical compressive loads to NP Cells. Mitochondrial phagocytosis can reduce oxidative stress-induced senescence in NP Cells by recycling damaged mitochondria. Western blotting results showed that high pressure loading treatments downregulated the expression of PINK1 and LC3II/I and upregulated the expression of Parkin and P62. At the same time, mitochondria showed shrinkage and enhanced membrane density in NP Cells treated with high pressure loading. All of these indicate that high pressure loading leads to impaired mitochondrial phagocytosis. Moreover, the proportion of senescence in NP Cells was significantly increased under high pressure. High mechanical load accelerates aging of NP Cells by inhibiting mitochondrial function and increasing oxidative stress.

Periosteal proteins and the associated classical pathway (NF-κB) are previously known to play a very critical role in stress-related cellular pathology. Wu et al. explored the mechanism of the role of periosteal proteins and pathways in mechanical stress-induced NP Cells senescence (Wu J. et al., 2022). They detected the expression of periostin and NF-κB/p65 in mechanically stressed-induced senescent NP Cells, and the results suggested that it is not only transcriptionally upregulated by NF-κB/p65, but also promotes catabolism in NP Cells by activating NF-κB. Therefore, the authors suggest that abnormal stress can regulate the aging process of NP Cells through periosteal proteins and NF-κB positive and negative feedback pathways.

What’s more, the Piezo1 ion channel in IVD Cells senses changes in mechanical stress and converts mechanical signals into chemical signals, which can also alert the process of IVDD (Zhao et al., 2018). Sheng et al. evaluated the role of Piezo1 in mechanical stress-mediated IVDD (Shi et al., 2022). Immunofluorescence staining results showed increased gene and protein levels of Piezo1 after high-intensity stress. In addition, qPCR and protein blotting results showed that high-intensity stress treatment also amplified the expression of classical senescence markers p53 and p16INK4a in NP Cells. Meanwhile, the investigators found that high-intensity stress not only increased the expression of inflammatory cytokines TNF-α, IL-6 and IL-1β, but also induced mitochondrial dysfunction by decreasing mitochondrial membrane potential and oxygen consumption rate in a time-dependent manner. Excessive mechanical stress increases pro-inflammatory cytokines by upregulating piezo1, which induces mitochondrial dysfunction and thus triggers aging of human NP Cells by inhibiting autophagy. Since Piezo1 is a non-selective cation channel and many studies have demonstrated that Ca2+ entry into Piezo1 promotes pro-inflammatory cytokine secretion and mitochondrial dysfunction, the authors speculate that Ca2+ influx plays a key role in Piezo1 signaling (Blythe et al., 2019; Yang et al., 2019).

#### 4.1.4 DNA damage

There are many mechanisms regarding DNA damage leading to aging, including through activation of signaling responses that block transcription and other DNA metabolism (Fagagna et al., 2003; Vermeij et al., 2016). DNA damage, as one of macromolecular damage, causes cells to undergo senescence is time-dependent accumulation (Yousefzadeh et al., 2021). Accumulated genomic and mitochondrial DNA damage causes stress-induced premature senescence (SIPS) in cells (Vo et al., 2016).

In IVDD, DNA damage is also involved in the aging process of NP Cells. It was found that DNA damage in IVD can promote aging by blocking the cell cycle and reducing the ability of cells to proliferate (Dimozi et al., 2015). The investigators used subcytotoxic concentrations of hydrogen peroxide (H2O2) to simulate oxidative stress and construct a model of DNA damage in NP Cells. Immunohistological results showed elevated expression levels of p53, p16INK4a and p21WAF1 in H2O2-induced senescent NP Cells, and pRb showed hypophosphorylation. Flow cytometry and SA-β-Gal staining results show that DNA-damaged NP Cells exhibit cell cycle arrest and an increased proportion of senescence. DNA damage leads to delayed G1 cell cycle and reduced cell proliferation through activation of the ATM-Chk2-p53-p21WAF1-pRb pathway. Nasto et al. found that rat with DNA defects were more susceptible to senescence of NP Cells and IVDD (Nasto et al., 2013). Researchers induced DNA damage with subtoxic doses of the chemotherapeutic agent mechlorethamine in wild-type and DNA repair-deficient Ercc1-/Δ mice. They found that mechlorethamine, an anticancer chemotherapeutic agent, accelerated the loss of ECM in IVD and greatly enhanced cellular senescence and apoptosis. And this effect was more pronounced in DNA repair-deficient Ercc1-/Δ mice than in Wt mice. These findings provide strong evidence that DNA damage can drive degenerative changes associated with IVDD even in normal hosts.

## 5 Anti-cellular senescence strategies

### 5.1 Reduction of senescent cells formation

To address the above triggering factors of aging in NP Cells, the following will summarize the therapies to relieve aging in NP Cells through four aspects, such as attenuating the inflammatory response, resisting oxidative stress and preventing mechanical stress, inhibiting DNA damage.

#### 5.1.1 Reduced inflammatory response

Inflammation as a driver of aging induction in NP Cells, current anti-inflammatory therapeutic studies in IVDD focus on inhibition of inflammatory pathways (e.g., NF-κB) and downregulation of inflammatory factors and enzymes (e.g., IL-1β, TNF-α).

NF-κB, a classical pathway of aging in NP Cells, can be used as a target for IVDD aging therapy. Huang et al. used a cationic polymer brush-coated carbon nanotube siRNA delivery platform to transduce LINC02569 siRNA to NP Cells and found that it significantly attenuated the IL-1β-induced inflammatory response and delayed the aging of NP Cells by blocking the NF-κB signaling pathway (Huang Y. et al., 2022). Xiaoliang Bai et al. assessed the role of higenamine in IL-1β-induced inflammation in human senescent NP Cells (Bai et al., 2019). The investigators isolated NP Cells from the IVD of IVDD patients and induced a mock inflammatory environment with IL-1β. By examination, higenamine was found to increase the cell viability of IL-1β-induced senescent NP Cells. Higenamine significantly attenuated the increased production of ECM degrading enzymes, as well as the IL-1β-induced production of a series of inflammatory factors such as nitric oxide synthase, nitric oxide, Prostaglandin E2, cyclooxygenase-2, TNF-α, and IL-2. Moreover, they examined the expression of IκBα, NF-κB, p65 and p-p65 using western blot. The results showed that higenamine attenuated IL-1β-induced p65 expression and NF-κB degradation in NP Cells. This indicates that higenamine also inhibits IL-1β-induced activation of the NF-κB signaling pathway in NP Cells, which is useful for inhibiting aging in NP Cells.

In Xin Huang’s study, IL-1β was used to mimic the inflammatory environment in IVDD, and it was found that the addition of Omentin-1 improved IL-1β-induced senescence in NP Cells, G1 phase cell cycle arrest and reduced ECM synthesis in NP Cells (Huang X. et al., 2022). These findings suggest that Omentin-1 plays an important function in protecting NP Cells against senescence and has the potential for IVDD gene targeting therapy. Li et al. explored the role of resveratrol in inflammation-induced senescence of NP Cells (Li et al., 2019). It was found that resveratrol partially reversed the effects of inflammatory cytokines (e.g., IL-1β, TNF-α) on the senescence of NP Cells, such as increased SA-β-Gal activity and ROS content, promotion of G0/1 cell cycle arrest, and gene/protein expression were upregulated. This suggests that resveratrol can effectively inhibit cellular senescence in an inflammatory environment. Qianchen Zhi et al. found that Dehydrolactone (DHE) partially attenuated TNF-α-induced ECM degradation and senescence of NP Cells (Chen et al., 2021). DHE is a natural sesquiterpene lactone isolated from medicinal plants with anti-inflammatory properties. Firstly, they investigated the role of DHE in TNF-α-induced activation of inflammatory signaling pathways and cellular senescence *in vitro*. Western blotting and SA-β-Gal staining showed that DHE inhibited the activation of NF-κB and MAPK inflammatory signaling pathways and ameliorated TNF-α-induced activation of STING-TBK1/NF-κB signaling-induced senescence in NP Cells. Subsequently, they measured the therapeutic effect of DHE in a mouse model of lumbar instability and found that DHE significantly attenuated the structural damage of the IVD, as evidenced by a significant reduction in the expression of TNF-α and IL-1β and the degradation of aggregated glycans within the IVD. The ability of DHE to alleviate TNF-α-induced ECM degradation and aging of NP Cells suggests that DHE is very promising for the treatment of IVDD.

#### 5.1.2 Resistance to oxidative stress

Oxidative stress as a risk factor for causing IVDD and aging inducing factor for NP Cells, therefore anti-aging treatment for oxidative stress in IVDD is necessary.

Shuo Zhang et al. found that heat shock protein 70 (HSP70) has a role in alleviating the aging of NP Cells in IVDD (Zhang et al., 2021d). They treated NP Cells with TBHP *in vitro* to mimic oxidative stress in NP Cells and used the HSP70 inducer TRC051384 to assess the cytoprotective effect of HSP70. SA-β-Gal staining and protein blotting analysis showed that HSP70 inhibited p53/p21-mediated senescence in NP Cells. And western blotting results showed that HSP70 could downregulate the JNK/c-Jun pathway (a pro-apoptotic and pro-inflammatory pathway). These indicate HSP70 impeded oxidative stress-induced senescence in human NP Cells by inhibiting the JNK/c-Jun pathway. Recently, goldenseal glycosides were found to inhibit TBHP-induced senescence and mitochondrial dysfunction in NP Cells by activating Nrf2, thereby inhibiting the deleterious effects induced by oxidative stress and enhancing the antioxidant capacity of NP Cells (Wang et al., 2019). In a similar vein, apigenin was found to protect NP Cells against TBHP-induced senescence and ECM degradation (Xie et al., 2022). Apigenin promotes nuclear translocation of transcription factor EB via AMPK/mTOR signaling pathway to alleviate TBHP-induced disruption of autophagosome-lysosome fusion and lysosome dysfunction, thereby protecting cells from oxidative stress (Liang et al., 2021; Xie et al., 2022).

SIRT1 has been shown to regulate cellular oxidative stress and can modulate the expression of p53 and p16 through deacetylation in NP Cells to mitigate the progression of aging (He et al., 2019). He et al. investigated the role and mechanism of action of SIRT1 in oxidative stress-induced senescence in rat NP Cells (He et al., 2019). The investigators used sublethal concentrations of H2O2 to induce senescence in rat NP Cells, and SRT1720 to activate SIRT1. Simultaneously, FoxO1 and Akt were inhibited by AS1842856 and MK-2206, respectively, to explore the role of the Akt-FoxO1-SIRT1 axis in rat NP Cells. Fluorescence quantitative PCR analysis showed that the protein expression of SIRT1 was gradually downregulated with increasing H2O2 concentration. Immunofluorescence revealed that the PI3K/Akt pathway was activated during this oxidative stress, and the activated phosphorylated Akt (Ser473) exerted post-translational modifications at the Ser1 locus and promoted FoxO1 phosphorylation. Activation of SIRT1 using SRT1720 showed downregulation reflecting senescence-related proteins (p53, p21, p16 and p-Rb), decreased expression of pro-inflammatory cytokines (TNF-α, IL-1β, IL-6 and IL-8), reduced G0/G1 phase arrest, and reduced proliferation and SA-β-Gal positive cells. SIRT1 ameliorated oxidative stress-induced senescence in rat NP cells, which is regulated by the Akt-FoxO1 pathway.

Zhang et al. found that Butein can be used as a therapeutic agent for diabetic IVDD patients by delaying the aging of NP Cells (Zhang et al., 2019). The investigators performed diabetic modeling in rats by intraperitoneal injection of streptozotocin. Senescence of NP Cells was measured by SA-β-Gal staining and found to be significantly increased in a dose-dependent manner after glucose treatment, and protein blotting results showed that high concentrations of glucose significantly promoted the expression of classical senescence-associated proteins p16INK4a and p21WAF1. Also increased acetylation of P53 and decreased expression of Sirt1 were found in both *in vivo* NP tissues and *in vitro* hyperglycemic NP Cells of diabetic patients. After the use of aging inhibitors of Sirt1, protein blotting results showed that the expression of Sirt1 was decreased and the acetyl-p53/p53 ratio was increased. This is in contrast to the results of butein action, indicating that butein can improve aging of NP Cells and IVDD in diabetic rats through the Sirt1/P53 axis.

Targeting mitochondria with oxidative stress dysfunction and applying antioxidants that target damaged mitochondria can help inhibit IVDD. Jianle Wang et al. found that Polydatin (PD) was able to reduce the level of NP Cell senescence by alleviating mitochondrial dysfunction (Wang J. et al., 2018). *In vitro*, NP Cells were pretreated with TNF-α to induce senescence while treated with PD. The results showed that PD reduced the number of SA-β-Gal-positive cells and the expression of p53 and p16 in TNF-treated NP Cells. Meanwhile, researchers collected NP tissues for histological and immunofluorescence analysis after 4 weeks of treatment with PD after puncture induction of IVDD in rats. Increased MitoSOX fluorescence of NP Cells in the TNF-α group suggested that TNF-α could exacerbate oxidative stress in NP Cells. However, MitoSOX fluorescence was significantly reduced in the PD group, indicating that PD promotes ROS clearance in NP Cells. In addition, the investigators examined the levels of Nrf2 in the nucleus. Immunofluorescence of Nrf2 showed that PD promoted Nrf2 translocation to the nucleus in a dose-dependent manner. Taken together, PD ameliorates IVDD in a rat model by promoting Nrf2 activity and inhibiting the senescence of NP Cells. Liang Kang et al. modeled IVDD by compression and found that MitoQ could significantly inhibit mitochondrial ROS production in NP Cells (Kang et al., 2020). Song et al. reported that protection of mitochondrial redox homeostasis by restoring the function of sirtuin 3 could attenuate IVDD (Song et al., 2018).

#### 5.1.3 Avoidance of excessive mechanical stress

Prevention of excessive mechanical stress is a promising way to slow down the process of IVDD. Actions with high IVD loads, such as torsion, bending, should be minimized or avoided (Walter et al., 2011; Chan et al., 2013). There is evidence that antibodies to periostin interrupt the PIEZO1-induced self-amplifying loop of NF-κB and periostin, delaying the production of senescent cells and may serve as potential therapeutic agents for IVDD (Wu J. et al., 2022). There is a lack of studies on the application of antibodies to periosteal proteins in IVDD models.

#### 5.1.4 Inhibition of DNA damage

It has been demonstrated that DNA damage as a driver of IVD aging significantly accelerates IVDD in mice (Wang et al., 2012). Despite the existence of complex repair mechanisms in cells, DNA damage continues to accumulate in NP Cells over time (Vo et al., 2016). Lack of gene repair mechanisms always leads to aging in multiple organ systems, such as IVDD (Hasty et al., 2003). It was found that DNA repair-deficient Ercc1/mice had an earlier onset of IVDD compared to normal mice, as evidenced by loss of proteoglycans, increased senescent NP Cells, and reduced disc height (Vo et al., 2010; Vo et al., 2013). Application of telomeric antisense oligonucleotides to a mouse model of accelerated senescence syndrome effectively reduced DNA damage response activation, senescence marker levels and SASP induction, and improved tissue homeostasis (Di Micco et al., 2021). The effectiveness of this method, although not confirmed in IVDD models, provides a research direction for the treatment of IVDD caused by cellular senescence due to telomeric DNA damage response. Apart from this, studies of exposed genotoxic stress in humans and mice have shown that DNA damage from ionizing radiation and smoking accelerates the development of IVDD (Nasto et al., 2013). Reducing or avoiding exposure to ionizing radiation and smoking therefore help to inhibit cellular aging and IVDD.

### 5.2 Removal of senescent cells

As one of the main modalities of ‘senotherapeutic’, ‘senolytics’ refers to pharmaceutical agents that can eliminate SCs. The combination of dasatinib (D) and quercetin (Q) has been better studied in recent years. And studies have reported that D can remove senescent adipose stem cells and Q can kill senescent endothelial cells and osteoblasts (Chen B. et al., 2022; Wu Y. et al., 2022). The combination of the D and Q can increase the content of proteoglycans in mouse IVD and is useful for eliminating SCs in CEP (Nasto et al., 2013; Chen B. et al., 2022). Emanuel J et al. prevented or treated age-related IVDD in 6-, 14-, and 18-month-old wild-type C57BL/6 mice targeted for senescent cells with weekly injections of a combination of D and Q. Histological analysis of lumbar discs from the 6- and 14-month D + Q cohorts showed significant reductions in aging markers such as p16INK4a and p19ARF and better preservation of tissue and cellular morphology, suggesting that D + Q treatment can effectively inhibit IVDD (Novais et al., 2021). The latest study has found that curcumin and its metabolite o-vanillin, a member of the “senolytics”, have been shown to eliminate SCs in IVD and to reduce IVDD (Cherif et al., 2019; Mannarino et al., 2021). Studies have demonstrated that the cell cycle suppressor p16INK4a is essential for the induction and maintenance of IVD senescence (Liu et al., 2019). Hosni Cherif et al. validated the drug’s ability to eliminate SCs by injecting RG-7112 into the central region of intact human IVD (Cherif et al., 2020). Immunohistochemical evaluation of p16Ink4a showed a significant reduction in the number of aging NP Cells after RG7112 treatment. Patil et al. found extensive reductions in both MMP13 expression and ECM degradation in NP after elimination of transgenic p16INK4a senescent NP Cells using ganciclovir (Patil et al., 2019a). This suggests that elimination of senescent NP Cells with high expression of p16INK4a can significantly slow down the aging process and reduce IVDD.

### 5.3 Others

Cell cycle arrest as one of the main features of cellular senescence, therefore inhibition of cell cycle arrest in senescent NP Cells is helpful for improving IVDD. Kunkun Sheng et al. measured the cell cycle of NP cells by flow cytometry, and β-galactosidase staining was used to study the senescence of NP cells. The results showed that p-coumaric acid significantly inhibited H2O2-induced cell cycle arrest and SA-β-Gal activity in a dose-dependent manner. Taken together, p-coumaric acid attenuated H2O2-induced oxidative stress and cellular senescence, suggesting a potential therapeutic use of p-coumaric acid in IVDD.

SASP is the main way in which SCs accelerate IVDD, so SASP inhibitors can prevent the cell-extrinsic effects of SCs(Di Micco et al., 2021). P16INK4a is considered to be a major regulator of SASP, and elimination of p16INK4a is able to reduce SASP(Novais et al., 2019). Activation of cannabinoid type 2 receptors inhibits p16INK4a protein and SASP, thereby restoring ECM metabolic homeostasis and reducing IVDD (Du et al., 2022). It was shown that TWIST1/2 protein supplementation also contributes to the partial reversal of the aging phenotype of NP Cells and facilitates the intervention of IVDD (Qiu et al., 2020). Both o-vanillin and curcumin effectively inhibited the SASP of NP Cells while eliminating senescent NP Cells, thus significantly increasing the synthesis of proteoglycans and Col II in NP(Cherif et al., 2019; Cherif et al., 2020). In addition, compounds that modulate NF-κB signaling, including metformin, apigenin, kaempferol, and BAY11-7,082, have also been shown to reduce SASP production, with apigenin reversing the imbalance between ECM synthesis and degradation by promoting the expression of Col II and proteoglycan and inhibiting the expression of matrix degrading enzymes (Di Micco et al., 2021; Xie et al., 2022). In conclusion, SASP inhibitors, unlike drugs that alleviate senescence-associated proliferative arrest, are particularly important in inhibiting senescent cellular intervention in the development of IVDD.

## 6 Discussion

IVDD is one of the leading causes of lumbar degenerative disease and has significant socioeconomic effects. SCs accumulate with age, leading to the normal aging process as well as age-related diseases. In healthy IVDs, the balance between anabolic and catabolic processes maintains ECM homeostasis; however, aging NP Cells develop SASP, which leads to an imbalance in expression between catabolic factors (e.g., MMPs and ADAMTS) and anabolic mediators (e.g., growth factors), ultimately leading to loss of ECM homeostasis and subsequent IVDD (Zeiter et al., 2005; Zhao et al., 2007; Sowa et al., 2008). Among the imaging manifestations of degenerative IVD, the NP, as the earliest and most widely degenerated structure, has the greatest impact on IVDD with its altered ECM component. Available studies have reported that DNA damage, inflammation, oxidative stress, and mechanical stress are all predisposing factors for IVDD, and all interact with aging. However, aging as one of the main causes of induction of IVDD, the initiating factors that cause NP Cells to undergo aging are still unclear. Therefore, in future studies on the correlation between aging and IVDD, more attention can be paid to the inducing factors and loci of action that trigger aging in NP Cells. Mitochondria are the main source of energy supply for IVD Cells and a major contributor to ROS production. Therefore, the stability of mitochondrial function is very important for IVD Cells. Mitochondrial dysfunction leads to oxidative stress, cell death and premature cellular senescence, all of which are associated with IVDD (Saberi et al., 2021). Studies have shown that depletion of dysfunctional mitochondrial sirtuins triggers cellular senescence (Wiley et al., 2016). Moreover, SCs are characterized by changes in mitochondrial mass, membrane potential and mitochondrial morphology (Chapman et al., 2019). Recent studies have shown that increased oxidative stress in SCs is associated with the accumulation of dysfunctional mitochondria (Di Micco et al., 2021). Mitochondria serve as a common site of action for a variety of IVDD causative factors, therefore mitochondria may be the focus of future IVDD research. Cellular senescence is mainly characterized by irreversible cell cycle arrest and SASP, and several drugs are available to treat IVDD by eliminating SCs and inhibiting SASP. This paper lists current anti-aging therapies for IVDD by combining the aging etiology of IVD Cells in IVDD and classical pathways of aging such as mTOR, AMPK, NF-κB, Sirtuins, P16, and p53 (Table 1). Hopefully, it could provide a little help for future IVDD research.

**TABLE 1:** IVDD anti-aging treatment.

Author	Medicine	Mechanism of action	Signaling pathways of action	year
Huang et al. (2022b)	eRNA LINC02569	Reduced inflammatory response	NF-κB	2022
Bai et al. (2019)	Higenamine	Reduced inflammatory response	NF-κB	2019
Chen et al. (2021)	Dehydrolactone	Reduced inflammatory response	NF-κB/MAPK, STING-TBK1/NF-κB	2021
Zhang et al. (2021d)	TRC051384	Resistance to oxidative stress	P53/p21, JNK/c-Jun	2021
Wang et al. (2019)	Goldenseal glycosides	Resistance to oxidative stress	Nrf2	2019
Xie et al. (2022)	Apigenin	Resistance to oxidative stress	AMPK/mTOR	2022
He et al. (2019)	SRT1720	Resistance to oxidative stress	Akt-FoxO1-SIRT1	2019
Zhang et al. (2019)	Butein	Resistance to oxidative stress	Sirt1/P53	2019
Wang et al. (2018a)	Polydatin	Resistance to oxidative stress	Nrf2	2018
Wu et al. (2022a)	Antibodies to periostin	Avoidance of excessive mechanical stress	NF-κB	2022

## 7 Conclusion

In conclusion, in this article, we discuss the etiology of IVDD and highlight the relevant role of senescent NP Cells on IVDD. The SASP of senescent NP Cells leads to an imbalance in IVD metabolic homeostasis and subsequently causes alterations in ECM composition in NP as a major cause of IVDD, and we describe in detail the mechanism of its role in IVDD. In addition, we listed some classical pathways that cause cellular senescence and anti-aging IVDD therapies, expecting to provide some help for the future clinical diagnosis and treatment of IVDD. Meanwhile, as SCs have been shown to be associated with multiple disease spectrums, studying SCs will also have a broad impact on the treatment of multiple systemic diseases.

## Abbreviations

IVDD, Intervertebral Disc Degeneration; NP, Nucleus Pulposus; LBP, Low Back Pain; IVD, Intervertebral Disc; ECM, Extracellular Matrix; AF, Annulus Fibrosus; CEP, Cartilaginous Endplate; Col II, Type II collagen; MMPs, Matrix Metalloproteinases; ADAMTS, A Disintegrin and Metalloprotease with Thrombospondin Motifs; SASP, Senescence-associated Secretory Phenotype; SCs, Senescent Cells; ROS, Reactive Oxygen Species; SA-β-Gal, Senescence-associated β-Galactosidase; Col Ⅰ, Type Ⅰ collagen; TBHP, Tert-butyl hydroperoxide; DHE, Dehydrolactone; HSP, Heat Shock Protein 70; PD, Polydatin; D, Dasatinib; Q, Quercetin; PCA, P-coumaric acid.
